# A New Approach to Visual-Based Sensory System for Navigation into Orange Groves

**DOI:** 10.3390/s110404086

**Published:** 2011-04-06

**Authors:** Joaquin Torres-Sospedra, Patricio Nebot

**Affiliations:** Department of Engineering and Computer Science, Universitat Jaume I, Avda sos Baynat S/N, E12071, Castellon, Spain

**Keywords:** image sensors, agricultural robotics, outdoor navigation, neural networks, ensembles

## Abstract

One of the most important parts of an autonomous robot is to establish the path by which it should navigate in order to successfully achieve its goals. In the case of *agricultural robotics*, a procedure that determines this desired path can be useful. In this paper, a new virtual sensor is introduced in order to classify the elements of an orange grove. This proposed sensor will be based on a *color CCD camera with auto iris lens* which is in charge of doing the captures of the real environment and an ensemble of *neural networks* which processes the capture and differentiates each element of the image. Then, the *Hough’s transform* and other operations will be applied in order to extract the desired path from the classification performed by the virtual sensory system. With this approach, the robotic system can correct its deviation with respect to the desired path. The results show that the sensory system properly classifies the elements of the grove and can set trajectory of the robot.

## 1. Introduction

In agricultural robotics, as in other mobile robotic systems, one of the most important parts is the navigation module. This part of the system is in charge of determining the optimal path the robot must follow in order to successfully perform a task (*i.e.*, spraying or weed detection). For instance, this path is located between two orange tree lines in the case of a robotic system which navigates into an orange grove.

Reviewing the bibliography, it can be seen that most navigation systems presented up to now are using general sensors like laser or sonar for path planning [1]. However, the price of this kind of sensors is high and their accuracy tends to be low under some adverse weather or lighting. Sonars can be affected by wind and temperature and lasers can lose accuracy because of lighting. On the other hand, some other approaches are based on vision systems composed by one or more cameras in order to address the navigation issue [2,3].

In this paper, a new sensory system based on a *color CCD camera with auto iris control* and a *neural network classifier* is introduced. The camera captures a real image, the objects and elements located in front of the robot, from the orange grove. The auto iris control allows to automatically adjust the brightness of the capture and obtain the best picture in changing lighting conditions. The neural network system processes each element from the original image and determines its areas as *Sky*, *Land/Soil*, *Orange trunks* and *Orange Crown*.

The advantage of this proposed sensory system is that the classifier (ensemble of neural networks) is flexible and provides good generalization ability [4–7]. Although the basic structure of an orange grove is well defined, its elements can slightly vary in a “new” grove. Another advantage is given by the fact that a neural network can be trained to perform the classification in different weather and lighting conditions.

In order to generate a classifier with high performance, an ensemble of neural networks has been generated. An ensemble is a set composed by plural neural networks which are applied to solve the same classification problem. They have been considered for this application because it has been demonstrated that an ensemble composed by some uncorrelated networks (the individual networks do not commit the same errors) provides better generalization ability than a single network [5,6]. Among all the alternatives to generate ensembles [8–16], *Simple Ensemble* has been chosen because it is simple, improves the accuracy with respect to a single network and does not require any previous complex study.

In this paper, the proposed navigation system is used to test that the information provided by the sensory system (composed by a single camera and a basic ensemble) is useful and accurate. The output of the sensory system is a *classification* image in which each pixel stand for a class label. From this image, the center of the path is calculated by using the *Hough Transform*. Finally, the desired path is obtained by extracting seven control points from the center of the path and using a 6*^th^* degree *Bézier curve*.

This paper is organized as follows. In Section 2, the classification tasks performed in the sensory system will be described. This part involves the capture of the real scenario and all the steps carried out to perform its classification. In Section 3, the whole process to detect the path that the robot should follow with the information provided by the sensory system will be shown. This section involves all the steps done to calculate the final trajectory. The experimental setup and some results are shown in Section 4.

## 2. Details of the Sensory System

### 2.1. Vision System

**Figure 1.**
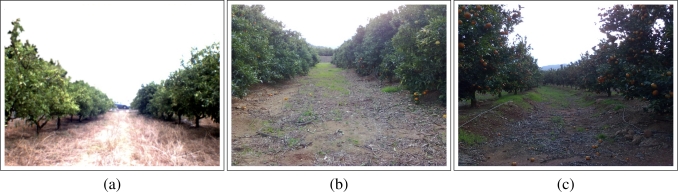
Image captured at an orange grove placed in Castellón (Spain).

The vision system considered for the application introduced in this paper is based on a color camera with *VGA* resolution. Some experiments were successfully performed using a normal camera with these features. In Figure 1, three different captures are shown.

In Figure 1(a), it can be seen that there are some orange trees at the left and right sides of the image. The land is located at the bottom of the image and the sky is at the top. This basic structure of the orange grove tends to be similar in different orange groves as it can be seen in Figures 1(b) and 1(c). However, it can be seen in the pictures that these groves are not “identical”. For instance, the orange crowns overlap the trunks in Figure 1(b) and the orange rows are not linear in Figure 1(c).

Moreover, the lighting conditions are different in the three images because the first one was taken in summer and the other two images were taken in winter with one hour gap between them.

Furthermore, the texture of the elements is different in each image. The soil in Figure 1(a) is quite different with respect to the other two images and the orange crowns in Figure 1(c) are plenty of oranges.

For these reasons, an accurate vision system is required for its use in the robotic system. Two different cameras with the same resolution were tested, a *logitech USB* web camera and a *FOculus* camera. Although the second alternative is much more expensive, it has been used in the experiments because the captured images are clearer even for those elements that are far from the camera.

Although better images are obtained with this kind of camera, the problem related to lighting conditions is not avoided. According to some suggestions done in the literature [17,18], the camera should allow the automatic iris control because the autonomous robot should navigate in an outdoor scenario, an orange grove, where the lighting conditions can vary. For this reason, this feature has been considered in the vision system used.

The camera used for our sensory sensor is a *FOculus FO124IC* and a *Cosmicar-Pentax* lens of 6 mm.

### 2.2. The Classification System

#### The Multilayer Feedforward Network

**Figure 2.**
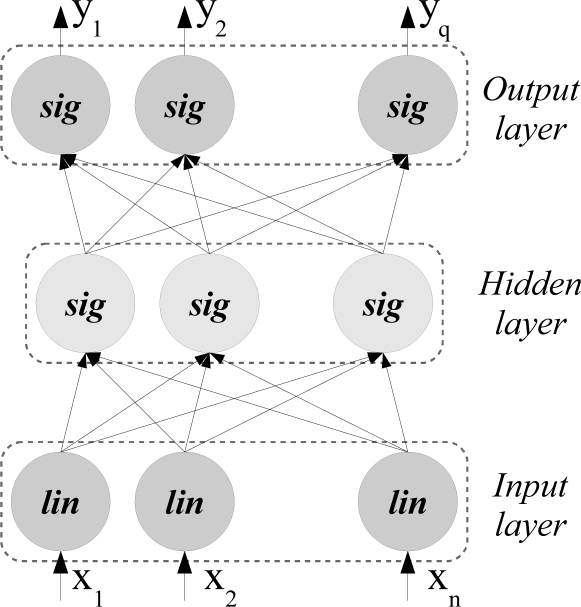
Multilayer feedforward Network.

The network architecture used in the experiments performed in this paper is the *Multilayer Feedforward Network*, henceforth called *MF network*. This network consists of three layers of computational units as it can be seen in Figure 2. The neurons of the first layer apply the identity function whereas the neurons of the second and third layers apply the sigmoid function. Furthermore, the connections between the input layer and the hidden layer, and the connections between the hidden layer and the output layer are weighted. These weighted connections will be denoted as *wih_i,j_* and *who_j,k_* respectively.

This kind of networks can approximate any function with a specified precision according to [4,7]. For this reason, it has been applied to classify real captures from an orange grove.

For the input layer, the input values, *ii*, and the output values, *io*, correspond to the value of the input parameters because the identity transfer function is applied in the input layer units.

(1) $${\mathrm{ii}}_{i}={\mathrm{io}}_{i}=x_{i}$$

For the hidden layer, the output values, *ho*, are given by Equation 2.

(2) $${\mathrm{ho}}_{j}=\frac{1}{1+\text{exp}\left(-\sum_{i=1}^{\mathrm{Ninputs}}{\mathrm{wih}}_{i,j}\cdot x_{i}\right)}$$

For the output layer, the output values, *oo* (also denoted by *y*), are given by Equation 3.

(3) $$y_{k}={\mathrm{oo}}_{k}=\frac{1}{1+\text{exp}\left(-\sum_{j=1}^{\mathrm{Nhidden}}{\mathrm{who}}_{j,k}\cdot {\mathrm{ho}}_{j}\right)}$$

where

- *N_inputs_* is the number of input parameters of the problem.
- *N_hidden_* is the number of units of the hidden layer.
- *N_classes_* is the number of classes of the problem.
- *wih_i,j_* corresponds to the value of the weighted connection between neuron *i* of the input layer and neuron *j* of the hidden layer.
- *who_j,k_* corresponds to the value of the weighted connection between neuron *j* of the hidden layer and neuron *k* of the output layer.

A learning process, also called training, has to be applied to establish the appropriate values of the connection weights. The first step sets random values to these weights. Then an iterative training algorithm is applied in order to adjust the weights and make them to converge to an “optimal” status. Usually, these training algorithms are based on gradient descend.

The whole process of training a *MF* network is described in Algorithm 1. In each epoch, the weights of the network have been adapted with the *Backpropagation* algorithm by using all the patterns from the training set, *T*. At the end of the epoch the *Mean Square Error*, *MSE*, has been calculated by classifying the patterns of the Validation set, *V*. When the learning process has finished, the weights of the epoch with lowest *MSE* of the validation set are assigned to the final network.

**Algorithm 1:** MF Network Training{*T*, *V*}

Set initial weights randomly
**for***e* = 1 to *N_epochs_***do**
**for***i* = 1 to *N_patterns_***do**
Select pattern *x_i_* from *T*
Adjust the trainable parameters with *x_i_*
**end for**
Calculate *MSE* over validation set *V*
Save epoch weights and calculated *MSE*
**end for**
Select epoch with lowest *MSE* and assign it as final net

To perform the experiments, the original datasets have been divided into three different subsets. The first set is the training set, *T*, which is used to adapt the weights of the networks. The second set is validation set, *V*, which is used to select the final network configuration. Finally, the last set is the test set, *TS*, which is applied to obtain the accuracy of the network. In the experiments introduced in this paper, the training and validation sets contain the same number of patterns selected from a reduced database and some results based on a small test set are shown.

#### Simple Ensemble

The process of designing an ensemble of neural networks consists of two main steps. In the first step, the development of the ensemble, the networks are trained according to the specifications of the ensemble method. The second step, the determination of the suitable combiner, focuses on selecting the most accurate combiner for the generated ensemble.

As has been previously described, the learning process of an artificial neural network is based on minimizing a target function (*MSE* in *Backpropagation*). A simple procedure to increase the diversity of the classifier consists of using several neural networks with different initial weight values.

Once the initial configuration is randomly set, the network can be trained as a single network. With this ensemble method, known as *Simple Ensemble*, the networks of the ensemble converge into different final configurations [19], therefore diversity and performance of the system can increase. The description of *Simple Ensemble* is shown in Algorithm 2.

**Algorithm 2:** Simple Ensemble {*T*, *V N_networks_*}

**for***i* = 1 to *N_networks_***do**
Generate a random seed value: *seed_i_*
Original Network Training {*T*, *V*}
**end for**
Save Ensemble Configuration

Then, the output of the networks are averaged in order to get the final output of the whole system. This way to combine an ensemble is known as *Output average* or *Ensemble Averaging*.

#### Codification of Captured Images for its Classification

A neural network, and any classifier, requires a set of inputs which are processed in order to obtain a final prediction. Selecting the most appropriate inputs is an important step because a neural network can provide better performance if they define well the classification problem. For terrain classification in orange groves, the features suggested in [20] for general terrain classification were used as inputs of the neural networks used in the ensemble.

The features used as inputs in the ensemble are calculated as follows. Firstly, the two-level *Daubechies wavelet transform—“Daub2”* is applied to each *HSI* channel from the image provided by the FOculus camera. With this procedure, seven sub-band images are obtained for each channel. Then two features, *Mean* and *Energy* are calculated with the pixels of the sub-band images which correspond to the *N × M* area of the captured image. Although this procedure provides 42 inputs features (1. The image contains 3 channels. 2. For each channel 7 sub-band images are generated with wavelets. 3. Two values are calculated for each subchannel) only 24 of them were used due to the noise introduced by the discarded ones. In the first experiments, these 24 features will be also used to perform the classification along with the two spatial coordinates (horizontal an vertical axes).

In this way, a pattern (element of the captured image) is represented by a 26-dimensional vector which is processed by each network of the ensemble in order to provide its classification.

#### Generating the Output Image of the Sensory System

**Figure 3.**
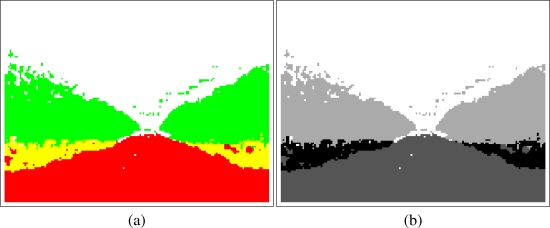
Classification image in color and gray scale representations.

The proposed sensory system provides an “artificial” image which represents the elements of the captured image. The classification is done block by block so the resolution of this image is lower than the original capture. An example of this “classification image” is shown in Figure 3.

In the classification image [Figure 3(a)] the colors are organized as follows: red refers to *Land/Soil*, white is *Sky*, green is *Orange crown* and yellow refers to the area of *Orange trunk*. It can be seen in the example that the classification is accurate and the important areas are correctly classified. Moreover, the basic structure of the orange grove can be easily seen in the classification image. Although this classification image is representative for humans, the procedures used to calculate the desired path required a gray scale image [Figure 3(b)].

Depending on the application, the sensory system will provide a *color* or a *gray scale* image. For instance, the color image can be useful for debugging or testing the classifier.

#### 3. Path Planner for Navigation in Orange Groves

The sensory system which provides the classification image has been introduced in the second section of the paper. Moreover, two representations of the classification image has also been show.

For this application, path planning, the gray scale representation has been selected for classification images because some specific image processing algorithms that have been applied requires this kind of images. Furthermore, the four gray colors shown in Figure 3 have been selected in order to maximize the contrast between classes and improve the performance of the whole system. This application is split into the following steps:

- Filtering the classification image.
- Detection of border between classes.
- Establishing the center of the path line.
- Determining the path by which the robot should navigate.

### 3.1. Filtering the Output Image

Although the classification obtained with the sensory system is accurate, some errors and noisy predictions are located in the boundaries between objects (classes). For this reason, a filtering stage has been introduced.

First of all, all the isolated blocks are removed. The class associated to a block is reassigned if it is not surrounded by another block of the same class. With this procedure some errors (and noise) located near the borders between classes are removed from the classification image.

**Figure 4.**
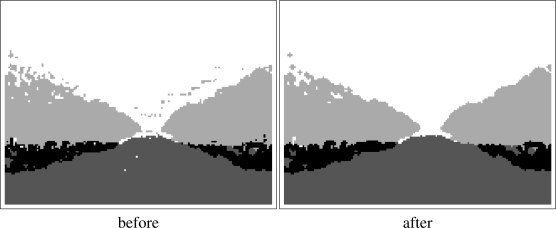
Classification image before and after filtering.

Then, an erode/dilate filter is used to remove small wrong areas. With this procedure, some errors which were not removed with the previous filter are now eliminated from the classification image. Moreover, the filter to eliminate all the isolated blocks can be applied again after the erode/dilate processing. An example of the classification image before and after the filtering is shown in Figure 4.

These filters have been selected because they are fast, an important feature in real time systems, and they successfully clean classification image by the sensory system. This filtering stage is “specific” in the procedure used to determine the desired path. For this reason, it was not introduced in the sensory system. After filtering the classification image, it is ready to be processed and extract the path.

### 3.2. Detecting Borders and Lines

With the filtered classification image, the path can be determined by detecting the main lines between the land and each orange tree row.

For this detection, an edge detector is used to obtain the blocks which correspond with the borders between two different classes in the filtered classification image. After some experiments, “canny” was chosen as edge detector.

**Figure 5.**
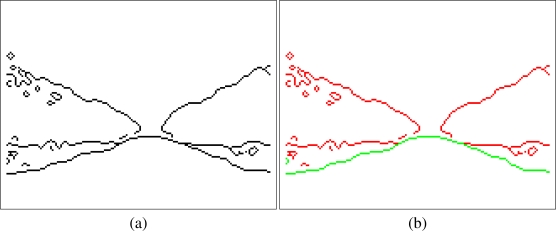
Borders (boundaries lines) between classes of the classification image.

Figure 5(a) shows the borders obtained after applying “canny”. At first sight, the five representative lines which represent the borders between classes can be seen. Among these five lines, only two of them (highlighted in green in Figure 5(b)) have to be extracted. These two lines represent the boundaries between Soil/Land and the orange trees. Although the other lines (highlighted in red in (b)) are also important they are not used at this stage of the system.

**Figure 6.**
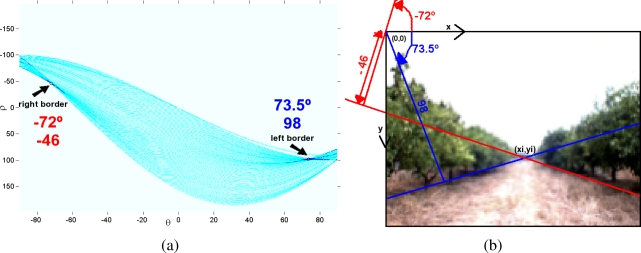
Hough transform of the classification image.

Then, the *Hough transform* is calculated using an image where only the borders between land and orange trees are represented (the green pixels in Figure 5(b)). In this procedure, the *Hesse standard form* (Equation 4) is used to represent lines with two parameters, *θ* (angle) and *ρ* (displacement). Each element of the resulting matrix of the Hough Transform contains a number of votes which depend on the number of the border pixels (denoted in green in Figure 5(b)) that are on the line. Figure 6 shows a graphical representation of the Hough Transform.

(4) $$y\cdot \sin\left(\theta_{\mathrm{path}}\right)+x\cdot \cos\left(\theta_{\mathrm{path}}\right)-\rho_{\mathrm{path}}=0$$

In Figure 6(a) a representation of the Hough’s matrix is shown where light blue stands for the lines with low votes and dark blue are assigned to the lines with highest vote values. According to the structure of the groves, the right border is represented with a negative angle whereas a positive angle is related to the left border. For this reason, one line with positive angle and one line with negative angle are selected from the Hough’s matrix according to their values. Concretely, the “positive” and “negative” lines with highest number of votes are extracted. In Figure 6(b) these two lines are drawn over the original capture. Moreover, the normal of those lines that passes over the center of coordinates (0,0) is also drawn because the parameters of the Hesse equation (angle and displacement) are related to it.

**Figure 7.**
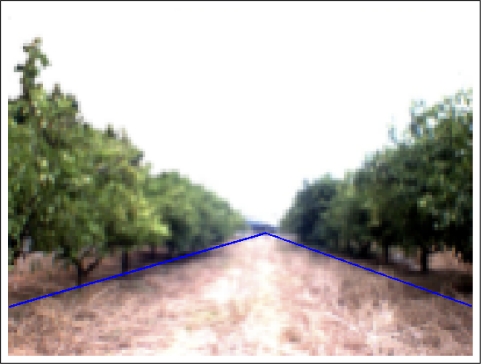
Detected border lines plot over the capture.

Finally, the borders of the grove are represented by the section of these lines located “under” their intersection, (*xi, yi*), as shown in Figure 7. These sections correspond to those points of the lines whose values of *x* and *y* are inside the image and whose *y*-value is higher than *yi*.

### 3.3. Establishing the Center of the Grove

**Figure 8.**
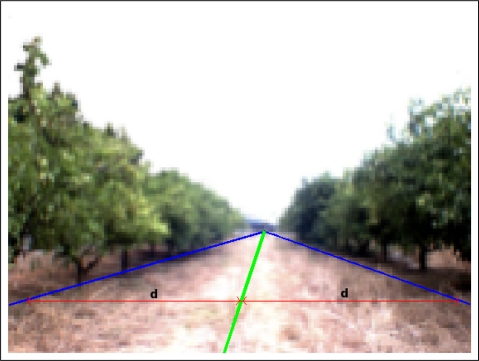
Example image with the representative lines (borders and path).

Once the two representative lines are extracted, borders between land and the two orange tree rows, the path is calculated. Concretely, the center of the path corresponds to the pixels located between these two lines. It means that the distance of the left border to the center of the path is the same as the distance from the right border to the same center for each point of the vertical axis. Figure 8 shows the original image of an orange grove where the borders between land and the orange trunks (blue line) and the center of the path (green line) are plotted over it. Red lines stand for the distance between the left and right borders with respect to the center of the path.

With the calculated center of the path and the information provided by other sources (*GPS*, *compass* or other maps) the robot can calculate intermediate points to correct its trajectory and navigate itself in the orange grove.

### 3.4. Improvement of the Precision in the Process of Establishing the Borders and the Path’s Center

In the image shown in Figure 8, it can be seen that the real left and right borders can not be represented by a simple line. For this reason, the area in which these borders are located has been vertically split into *n* different subareas. After performing some experiments, *n* has been set to value 5.

**Figure 9.**
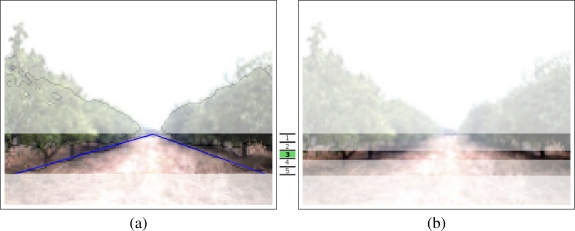
Example image with the representative borders with the area of interest and an example of subarea. The images are artificially generated from Figures 5 and 6.

**Figure 10.**
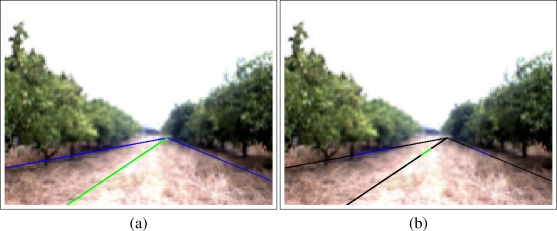
Example representative borders lines for the third subarea.

The area of interest and an example of subarea (concretely the third subarea) are shown in Figure 9 whereas Figure 10 shows the border lines for the subarea shown in the example.

Then, the two border lines have been calculated using the information provided only in the corresponding subarea. When the two border lines are calculated, the center of the path can be extracted as done with the general borders. Figure 10(a) shows the border lines for the third subarea.

At first sight it can be noticed that they are different with the general borders shown in Figure 7 because less information (only the border pixels from the third subarea) has been used to calculate them. The new lines represent better the borders and the center of the path for the concrete subarea whereas the lines shown in the previous subsections represent the borders and center of the path in a more general way. Moreover, the lines calculated for a subarea may not represent well the whole border as can be noticed in the figure. In fact, only the part of the line located in the pixels that belongs to the subarea (colored accordingly in Figure 10(b)) will be used whereas the other parts of these lines (colored in black in Figure 10(b)) will not be used.

**Figure 11.**
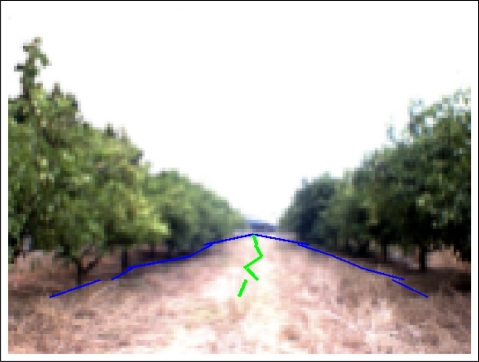
Example image with the representative borders for all the subareas.

Once the representative lines (borders and centers of the path) are calculated for all the subareas, a better representation of the complete borders and center of the path are obtained. In Figure 11, the borders and center of the path are shown for all the subareas.

### 3.5. Establishing the Desired Path for the Robotic System

In this last stage of the path planning, the desired path by which the robot should navigate is established. In this case, the trajectory is calculated from the bottom center of the image (where the robot is placed) to the center of the path and then continuing moving centered.

A Bézier curve can be calculated using the bottom center of the image and the intersection between the main two border lines using two intermediate control points. However, this approach does not take benefit from the optimization performed in subsection 3.4.

**Figure 12.**
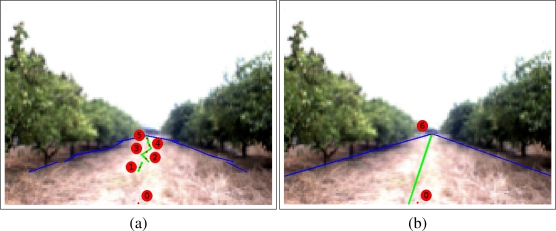
Visualization of the seven control points used for establishing the trajectory.

To use the optimization performed, a Bézier curve of 6*th* degree is used to calculate the final trajectory. The first point, *P*_0_, is given by the bottom center of the image because the image is centered with respect to the robot. The following five points (control points), *P*_1_ to *P*_5_, are given by the center of the path calculated in each subarea of the image. Concretely, the middle point of each green line is chosen as a control point. The last point, *P*_6_, is given by the intersection between the two general border lines. All the control points, *P_i_*, correspond to their coordinates on the image (*x_i_, y_i_*). Figure 12 shows an example of the visualization of these control points.

With these seven control points, the trajectory of the robot will be approximated using Equation 5. This equation is used to calculate the 6th degree *Bézier curve* with the *Horner’s rule* for efficient computation.

(5) $$B\left(t\right)=\left(\left(\left(\left(\left(c_{6}\cdot t+c_{5}\right)\cdot t+c_{4}\right)\cdot t+c_{3}\right)\cdot +c_{2}\right)\cdot t+c_{1}\right)\cdot t+c_{0},t\in [0,1]$$

Where the coefficients are:

(6) $$c_{0}=P_{0}$$

(7) $$c_{1}=6\cdot \left(P_{1}-P_{0}\right)$$

(8) $$c_{2}=15\cdot \left(P_{0}+P_{2}-2\cdot P_{1}\right)$$

(9) $$c_{3}=20\cdot \left(3\cdot \left(P_{1}-P_{2}\right)+P_{3}-P_{0}\right)$$

(10) $$c_{4}=15\cdot \left(P_{0}+P_{4}+2\cdot \left(3\cdot P_{2}-2\cdot \left(P_{1}+P_{3}\right)\right)\right)$$

(11) $$c_{5}=6\cdot \left(P_{5}-P_{0}+5\cdot \left(P_{1}-P_{4}+2\cdot \left(P_{3}-P_{2}\right)\right)\right)$$

(12) $$c_{6}=P_{0}+P_{6}+3\cdot \left(-2\cdot \left(P_{1}+P_{5}\right)+5\cdot \left(P_{2}+P_{4}\right)\right)-20\cdot P_{3}$$

**Figure 13.**
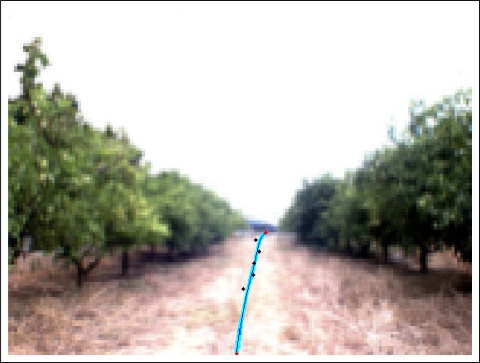
Example of the calculated trajectory.

Figure 13 shows the original capture. In the image, the trajectory (by which the robot should navigate) is drawn on it with light blue.

## 4. Experimental Setup and Results

### 4.1. Experiments

To perform this first study a simple ensemble of *MF* networks has been generated using a dataset with a few test images.

#### Network Parameters

The *MF* networks used in the experiments use the following basic structure:

**Table:** 

*Input parameters*:	26
*Hidden units:*	10
*Output classes*:	4

The parameters of *Backpropagation* used to adapt weights of the networks are the following ones:

**Table:** 

*Adaptation Step*:	0.1
*Momentum rate*:	0.05
*Number of iterations*:	1500

Finally, nine networks have been trained with the previous parameters in order to generate the ensemble. This value, 9 networks, has been chosen because *Simple Ensemble* reports good performance for this size according to [21,22].

#### Block Image Parameters

In the experiments carried out in this paper, the block size have been set to 8 × 8 after testing some different sizes (4 × 4, 8 × 8, 16 × 12, and 16 × 16). With this block size the performance of the ensemble is good enough (as shown with the example classification image) and the resolution of the classification image is not low.

Furthermore, the resolution of the classification image, where a pixel corresponds to the classification of the corresponding image block, should have been 80 × 60 for VGA captures and 8 × 8 blocks (640/8 = 80 and 480/8 = 60). In this case, the distance between blocks corresponds to the block size and there is not any overlap between blocks. However, the resolution of the classification images provided by the introduced sensory system is 159 × 119, almost doubled in each axis. To increase the resolution, the distance between blocks have been reduced by 50% and overlapping between two connected image blocks is allowed.

### 4.2. Results

**Figure 14.**
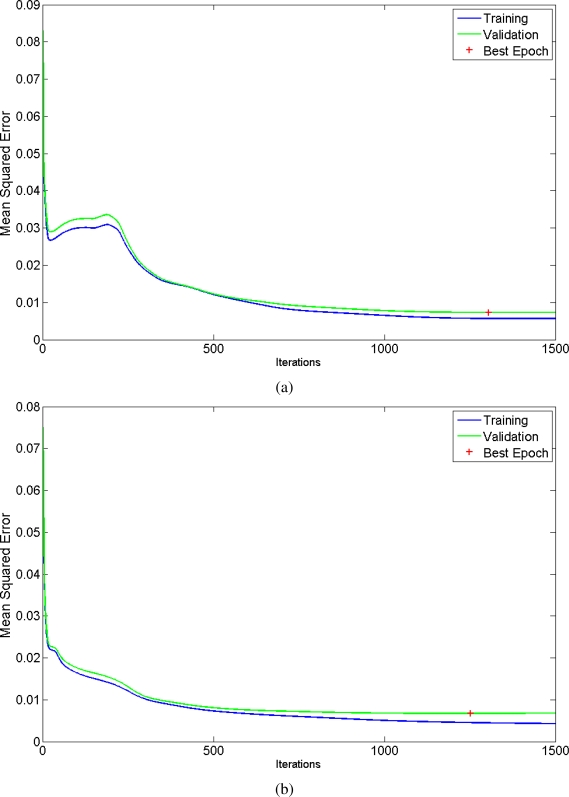
Training curve of two networks (MSE *vs.* Iteration time).

The generated ensembles have been tested with some captures in order to test the sensory system in path planning. First of all, the training curve of two networks of the generated ensemble are depicted in Figure 14. The green lines denote the *Mean Squared Error* calculated with the patterns of the validation set. Similarly, the blue lines correspond to the *MSE* related to the training patterns. The best epoch, the one used to select the final parameters of the network, is denoted with the red cross.

**Figure 15.**
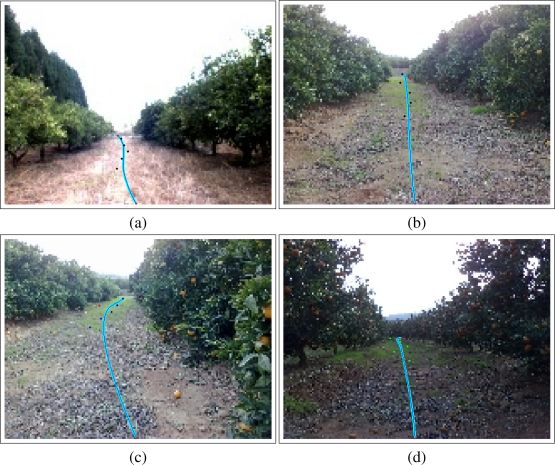
Some results and the calculated trajectories.

Then, Figure 15 shows four captures with the desired path (by which the robot should navigate) drawn on it. These images were taken in different groves with different environmental (lighting and weather) and positional conditions.

In these four figures, it can be seen that the path (drawn with light blue) is properly calculated. On the one hand, Figures 15(a) and 15(b) (captures done in “normal” groves) show that the robot is slightly displaced. These images are related to the typical captures that will be processed by the system and application introduced in this paper. On the other hand, two examples are shown with those cases in which the orange rows are not linear (Figure 15(c)) or the robot is highly displaced (bottom right image). Moreover, Figure 15(d) also shows the correct path in a “dark” capture.

## 5. Conclusions

In this paper, a new sensory system based in a *color CCD camera with auto iris control* and a *Simple Ensemble* of neural networks has been introduced. This new system is introduced to capture images from an orange grove and provide the classification of the elements it contains.

With the classification image, some representative lines are extracted with the Hough transform and the center of the path is established with them. This procedure is applied to the whole image and in five representative subareas of the capture. With these lines, calculated in general and for the five subareas, the final trajectory is set with a Bézier curve of 6th degree.

The results shown in this paper are promising. The desired path is properly calculated in different lighting conditions. The desired path is calculated with the classification provided by *Simple Ensemble*. This classifier provides nearly 95% of correctly classified patterns. Moreover, the determination of the center of the path in some subareas has had a positive effect in those groves whose structure is not linear because the center of the path is also properly obtained.

Finally, some further work will be focused on optimizing the classification system. Although the performance of *Simple Ensemble* is high, some well-known ensembles such as *Bagging* [8], *Boosting* [9–12] and *Cross-Validation Committee* [13–16] will be tested on this concrete application. The best ones will be applied to improve the performance of the classifier as suggested in [21,22] for an heterogeneous set of classification problems.
